# Effect of Paste Fluidity and Paste-to-Aggregate Ratio on the Strength and Permeability of Porous Mortar from Manufactured Sand

**DOI:** 10.3390/ma15249065

**Published:** 2022-12-19

**Authors:** Weichao Li, Laibo Li, Yali Li, Yanlin Li, Lingchao Lu, Xin Cheng

**Affiliations:** 1 Shandong Provincial Key Laboratory of Preparation and Measurement of Building Materials, University of Jinan, Jinan 250022, China; 2 Centre for Smart Infrastructure and Digital Construction, School of Engineering, Swinburne University of Technology, Hawthorn, Melbourne, VIC 3122, Australia; 3 Tiezheng Testing Technology Co., Ltd., Jinan 250014, China

**Keywords:** manufactured sand-based porous mortar, mix proportion design, response surface method

## Abstract

Many places in the world suffer from a shortage of river sand because of population growth and environmental protection, and people have to replace river sand with manufactured sand (M-sand). In this study, M-sand was adopted as aggregate and the effect of the mix design (paste fluidity (PF) and paste-to-aggregate ratio (P/A)) on the properties of porous mortar was investigated through a combined experimental, statistical and response surface method (RSM). By including variations in both P/A (0.16–0.26) and PF (160–200 mm), the method was utilized to develop ANOVA models and construct response surface and contour lines. The experimental results revealed that the compressive strength of the porous mortar increased by 62.3% to a value of 34.1 MPa while the PF increased to 190 mm from 160 mm at a P/A of 0.20, and the water permeability coefficient was 7.2 mm/s under the same conditions. In addition, the ANOVA analysis of the measured properties revealed a strong interactive effect of the paste-to-aggregate ratio and paste fluidity on the porous mortar properties, and the developed relationship models between the variables and responses were accurate. A porous mortar with a compressive strength over 30 MPa and a permeability coefficient over 7 mm/s could be conveniently designed by RSM. Additionally, the compressive strength of the porous concrete reached more than 40 MPa at a P/A of 0.26.

## 1. Introduction

Porous concrete has recently attracted a considerable amount of attention as an eco-friendly construction material that is in harmony with nature [1,2,3]. Having a similar composition to conventional normal concrete [4,5], one most important feature of porous concrete is its large interconnected voids created by omitting fine aggregate [6]. These provide porous concrete with many beneficial characteristics and capabilities, such as water permeability, acoustic absorption, water purification, SOx and NOx adsorption, humidity control, and even functions related to vegetation [7,8,9,10,11]. Generally, porous concrete consists of an under layer and a surface layer, and aggregates account for 75–80% of the volume of the surface layer and would therefore be expected to have an important influence on the properties of porous concrete. The recent rapid growth in the porous concrete industry has raised a concurrently increased demand for aggregate [12]. For centuries, river sand has been extracted from river beds and used as fine aggregate. However, there have been severe global concerns over the reckless extraction and increasing scarcity of river sand due to the boom in infrastructure and, thus, massive demand for concrete [13].

At present, manufactured sand as a new alternative aggregate [14] has been widely investigated. However, one of the most hindering factors for the use of manufactured sand in porous mortar [15,16] is the absence of a specific mix design to obtain the desired fresh paste property and hardening performance. In general, porous concrete shows low strength due to its large void ratio. Fortunately, surface coating technology could effectively enhance the strength of porous concrete because the cement paste is well distributed around the aggregate by the same technology as described above [17]. For the strength of porous concrete, there is a significant effect from the cement paste fluidity and paste-to-aggregate ratio on this property. The work of Yan et al. suggested the paste-to-aggregate ratio determined the aggregate coating thickness and the cement paste fluidity determined the uniformity of paste coating on the aggregate surface [17]. In addition, Gong et al. proved that there is a direct relationship between the fluidity of cement paste and the water permeability coefficient of porous concrete due to the pores in the bottom of porous concrete being blocked when the fluidity of the cement paste was too high [18]. However, there are few methods to study the effects of the two factors. It is worth noting that the response surface method (RSM) can explore the influence of multiple factors on the same result. Montgomery [19] defined RSM as a mathematical and statistical technique used for designing experiments. The relationship between multiple factors can be established, the relevant conditions of the parameters can be optimized and the best response can be predicted by RSM [20]. For instance, Chen et al., utilizing RSM, determined the optimum selection of achieving sustainable and energy-saving building materials [21]. The work of Lu et al. suggested that RSM is efficient for studying the effect of admixtures on the properties (e.g., setting time, apparent viscosity, plastic viscosity) of sulphoaluminate cement paste [22]. For porous mortar, balanced water permeability and mechanical strength are desired.

In this work, M-sand was used instead of river sand as aggregate, and the relationship between the compressive strength and the permeability coefficient of porous mortar was established by RSM. The main aim of this research work was to develop a new generation of porous mortar using manufactured sand as fine aggregate through the RSM technique. Specifically, the synchronous effect of cement paste fluidity and paste-to- aggregate ratio on the porous mortar’s properties (e.g., compressive strength, void ratio and water permeability coefficient) was investigated using RSM and statistical analysis. Thereby, the optimal mix formulation for a porous mortar with the desired properties could be readily identified. The research findings generated in this study are expected to provide useful scientific data to advance porous mortar fabrication using manufactured sand.

## 2. The Review of Previous Studies

It is interesting to explore alternative materials with a technical performance comparable to that of existing aggregates: for instance, manufactured sand, steel slag, copper slag, recycled aggregates, etc. [23,24]. Among these, manufactured sand produced by crushing limestone, granite, basalt, etc., has been receiving great attention these days [25]. The main difference between manufactured sand and river sand lies in particle shape, surface roughness, grading, content of stone powder (micro fines), water adsorption capacity, etc. The properties (e.g., workability, water demand, mechanical properties and durability) of manufactured sand concrete were also observed to be different from those of river sand concrete [23,26,27]. A general observation in these studies presented the adverse effects of fine content, shape and texture on the workability of concrete, the elimination of which was attempted by increasing water/cement ratio, paste/aggregate ratio or using increased amounts of superplasticizer. In addition, improved compressive strength and durability was observed in the manufactured sand concrete. For instance, the work by Nanthagopalan and Santhanam [23] suggested that a relatively high paste volume was essential to achieve the required flow for self-compacting concrete using manufactured sand in comparison with river sand. The work by Donza et al. suggested that concrete with manufactured sand required an increase in superplasticizer use to obtain the same slump [28]. The study by Arulmoly et al. using manufactured sand to partially replace river sand in cement mortar, showed that the inflated bleeding, workable life, water retention, flexural strength, compressive strength, capillary water absorption, linear shrinkage and thermal expansion of mortars were affected with the selected replacement levels [29]. It was also reported that the concrete had better shrinkage and durability at proper gradation of manufactured sand, dosages of admixtures, appropriate mixing and placing approach [30]. Therefore, in spite of its useful hardening properties, the wide spectrum application of manufactured sand in concrete demands an in-depth and systematic study of the mix design to achieve balanced workability and hardening properties. 

## 3. Experimental Work

### 3.1. Materials

Ordinary Portland cement (OPC, Grade 52.5 R according to Chinese National Standards GB175-2007, manufactured in Shandong Cement Co., Ltd, China) was used as the cementitious material, and its initial and final setting times were 115 and 170 min, respectively. The chemical compositions of OPC were measured by X-ray fluorescence spectrometer (Tiger S8, Karlsruhe, Germany), and the results are shown in Table 1. Limestone manufactured sand (M-sand, from Changqing Commercial Concrete Co., Ltd., Chongqing, China) was adopted as aggregate for porous mortar, and its physical properties are listed in Table 2. Polycarboxylate polymer (PP, water-reducing ratio over 25%) was used as superplasticizer.

### 3.2. Mixture Design

Firstly, the effect of cement paste fluidity (PF) on the basic physical properties (e.g., compressive strength, void ratio and water permeability coefficient) of porous mortar was investigated with fixed ratios of water-to-cement (W/C) and paste-to-aggregate (P/A) 0.26 and 0.2, respectively. These ratios are commonly used for fabricating porous concrete [31]. Cement paste mixes with varied fluidity from 160 to 200 mm were obtained by adjusting the addition of PP (Table 3). Secondly, RSM was used as an optimization technique to investigate and optimize the synchronous effect of PF and P/A ratio on porous concrete properties. Sixteen mixtures with varied PF (160–200 mm obtained by adjusting PP addition) and P/A ratio (0.16–0.26 were prepared. W/C ratio remained at 0.26 for all the mixtures. Compositions of the mixtures are shown in Table 4. 

### 3.3. Specimens Preparation

The mixing procedure proceeded as follows: (a) M-sand, PP and mixing water were put into a mixer and mixed at 48 rpm for 30 s; (b) OPC was slowly added into the mixture with continuous mixing for 60 s; (c) the mixing continued for another 120 s. Then fresh porous mortar samples were cast in cuboid molds (40 × 40 × 160 mm) and cylindrical molds (φ 100 × 100 mm), and were consolidated by an electric vibrator for 30 s. The molded specimens were then cured (20 ± 2 °C, 95 + % RH) for 24 h. The specimens were then demolded and placed under water at 20 ± 2 °C for another 27 days. 

### 3.4. Measurements

The compressive strength of cuboid specimens was determined according to GB/T 50081-2003 [32]. Total and interconnected void ratios of cylindrical specimens were measured, referring to [31]. Specifically, at the end of the 28-day curing in water, the mass of the demolded specimens was measured: M_1_—initial mass of samples in the water; M_2_—final mass tested following air drying air-dried mass (25 ± 1 °C, 20 ± 2% RH) for 24 h; M_3_—final mass tested following air drying saturated air-cured mass (25 ± 1 °C, 95 ± 2% RH) for 24 h. The total void ratio (*R_TV_*) was then determined by Equation (1) and the interconnected void ratio (*R_CV_*) by Equation (2), where V was specimen volume and ρM was water density:

(1) $$R_{TV}=\left[1-\frac{M_{2}-M_{1}}{V\cdot \rho M}\right]\times 100\%$$

(2) $$R_{CV}=\left[1-\frac{M_{3}-M_{1}}{V\cdot \rho M}\right]\times 100\%$$

The water permeability coefficient (WPC) of porous mortar was measured in accordance with (ISO 17785-1, 20160). WPC of porous mortar was determined over a period of 60 s under a water head of 100 mm. The equation used to obtain WPC was as follows:

(3) $$\mathrm{Kr}=\frac{H}{h}\times \frac{Q}{A\left(t_{2}-t_{1}\right)}$$

where Kr: WPC (mm/s); H: length of specimen (cm); Q: amount of drain off water from *t*_1_ to *t*_2_ (cm^3^); h: difference of water head; *t*_2_-*t*_1_: time (s); A: area of cross section of cylindrical specimen (cm^2^).

SEM images were obtained using a field emission scanning electron microscope (FESEM, FEI, QUANTA FEG 250, Lincoln, NE, USA). The viscosity of cement paste was measured using a rheometer (Kinexus Lab, Malvern, UK). In order to stabilize the system, the paste was stirred at the speed of 50 s^−1^ and its viscosity was monitored continuously for a duration of 800 s.

### 3.5. RSM Modeling

RSM is a technique for collecting statistical analysis and mathematical methods to determine the relationships between several independent variables and one or more response variables [33,34]. By manipulating multiple inputs at the same time, RSM can identify important interactions that may be neglected when experimenting with one factor at a time. RSM involves the proper design of experiments to obtain the response data, polynomial model development and ANOVA analysis to validate the adequacy of the model which is then used to guide independent variable optimization for the desired response. Such a statistical method has been proven effective in helping researchers develop new products with less experimentation. In this study, RSM was adopted to examine the influence of paste fluidity (PF) and paste-to-aggregate ratio (P/A) on the properties of porous mortar and develop the appropriate mix proportion.

A commercially available software package (Design-Expert_9.0.0a) for experimental design and analysis was used to plan the experiment. The set-point design included two variables and two responses. In this study, an RSM model with a total of 16 set points was constructed. The entire data set, along with the corresponding experimental data, is presented in Table 4. Because compressive strength and WPC are important properties of porous mortar, their responses to mix design variables, i.e., PF, P/A, were considered to develop the RSM model. Once the experimental data were collected, a polynomial model was developed for each response. The analysis of variance (ANOVA), which is mainly used to determine the *p*-value and F-value, was performed to verify the adequacy of the model.

After confirming the model, ANOVA analysis was used to assess the influence of each alternating quantity on the responses. The importance of each parameter was determined by identifying whether a *p*-value is below the threshold value (usually 0.05), which indicates that the terms are significant and that their contribution improves the model. During the estimation, the model terms with a *p*-value higher than 0.05 were not deemed to be statistically significant and therefore were removed. The appropriate regression model, recommended by [34], was applied, as shown in the following equation:

(4) $$y\left(x\right)=b_{0}+\overset{n}{\underset{i=1}{\sum }}b_{i}x_{i}+\overset{n}{\underset{i<}{\sum }}\overset{n}{\underset{j}{\sum }}b_{ij}x_{i}x_{j}+\overset{n}{\underset{i=1}{\sum }}b_{ii}x_{i}^{2}+\overset{n}{\underset{i=1}{\sum }}b_{ii}x_{i}^{3}+\overset{n}{\underset{i\neq }{\sum }}\overset{n}{\underset{j}{\sum }}b_{ijj}x_{i}x_{j}^{2}\;+\overset{n}{\underset{i<}{\sum }}\overset{n}{\underset{j<}{\sum }}\overset{n}{\underset{k}{\sum }}b_{ijk}x_{i}x_{j}x_{k}+\varepsilon$$

where $y\left(x\right)$ is the response, $x_{i}$, $x_{j}$ and $x_{k}$ are the parameters, b is the regression coefficient, n is the number of parameters included in the experiment, and ε is the random error.

## 4. Results and Discussion

### 4.1. Effect of Cement Paste Fluidity on the Basic Physical Properties of Porous Mortar

**Figure 1:**
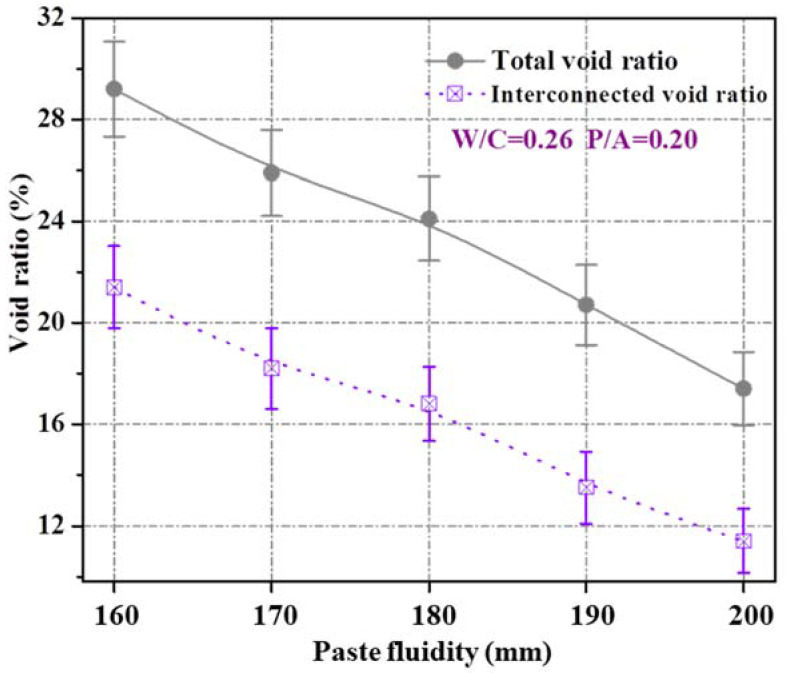
Impact of paste fluidity on void ratio of porous mortar.

Figure 1 presents the effect of cement paste fluidity on the void ratio (i.e., total and interconnected void ratio) of the porous mortar prepared by a fixed P/A 0.2. It was obvious that the total void ratio decreased linearly with the increase in cement paste fluidity. For instance, when the cement paste fluidity was 160 mm, the total void ratio reached 29.2%, and in cases of cement paste fluidity reaching 200 mm, the total void ratio decreased by 40.4% to a value of 17.4%. A possible reason was that the uniformity of the aggregate coating thickness (i.e., OPC paste coated over the manufactured sand surface) increased with the increase in cement PF. However, the very low void ratio at oversized cement paste fluidity (e.g., 200 mm) might be due to the detachment of the paste from the manufactured sand and accumulation in the bottom of the porous mortar during the process of consolidation [31]. The content of the valid interconnected pores is highly related to the air void content in the mortar mixture [35]; the statistics indicate that the interconnected void ratio decreases almost parallelly to the total void ratio, suggesting the portion of interconnected void volume in the total void volume remains unchanged despite the changes in PF. Therefore, in the following sections, only the total void ratio was used to explain the observed porous mortar properties.

**Figure 2:**
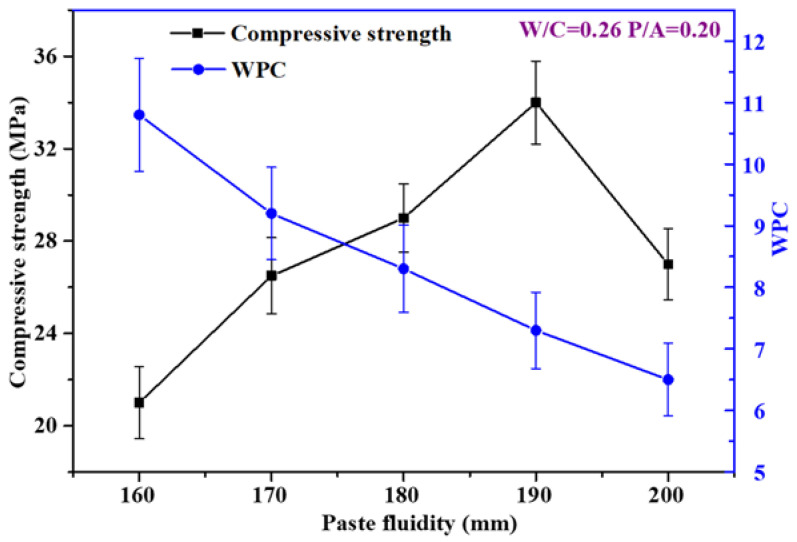
Relationship between compressive strength and paste fluidity (square solid line) and WPC and paste fluidity (bullet solid line) (Note: P/A was fixed at 0.20).

**Figure 3:**
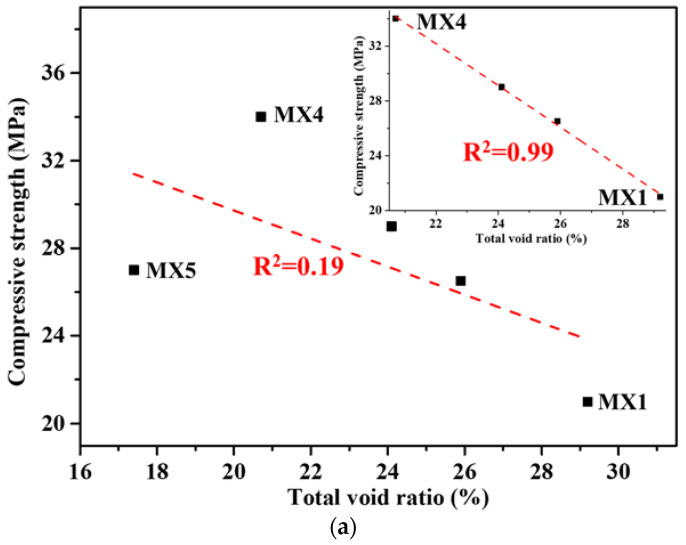

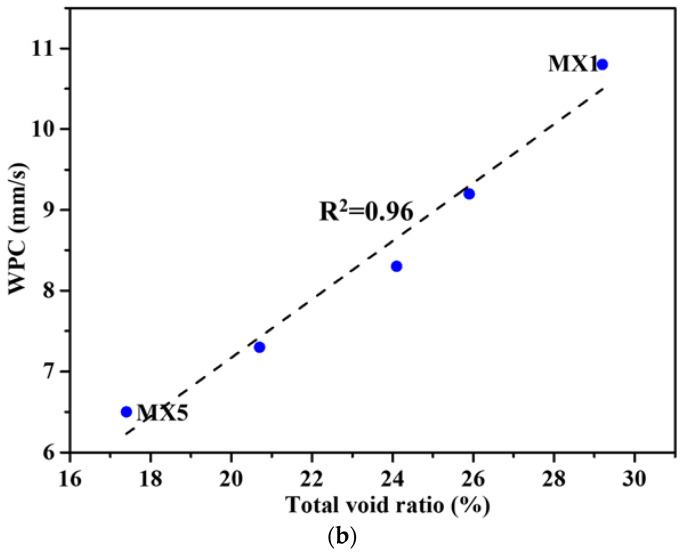
Correlation between (**a**) mean compression strength and total void ratio (inlet is the correlation neglecting MX5); (**b**) WPC and total void ratio.

The relationship between the compressive strength and WPC of the porous mortars and the paste fluidity prepared by a fixed P/A is shown in Figure 2. In the case of a paste fluidity of no more than 190 mm, the compressive strength of the porous mortar increased with the increase in paste fluidity. When the PF was 190 mm, the compressive strength increased by 62.3% to 34.1 MPa compared with that of a paste fluidity of 160 mm. However, in the cases of paste fluidity reaching 200 mm, the compressive strength decreased to 27.2 MPa. Generally, the compressive strength of the porous concrete mainly depended on its total void ratio [36,37]. The decrease in density and effective cross section caused by high porosity leads to the formation of a local tension zone around the pores and the promotion of local failure, resulting in the decrease in strength [38]. A possible reason was that the total void ratio of the porous mortar decreased with the increase in cement PF (no more than 190 mm). The relationship between the compressive strength and the total void ratio is shown in Figure 3a. An excellent linear correlation between the measured compressive strength and the total void ratio was observed for MX1 to MX4 (Figure 3a inlet). However, with the inclusion of MX5, such correlation disappeared (Figure 3a). This might be the result of when the cement paste fluidity reached 200 mm, the cement phase was not uniformly distributed in the porous mortar—a denser presence, thus a lower void ratio at the bottom of the porous mortar but less presence, thus a higher void ratio in the top portion of the mortar and overall reduced compressive strength. This also suggested that the compressive strength of the porous mortar depended on its total void ratio only when the cement paste evenly coated the aggregate surface. 

The WPC decreased with the increase in cement paste fluidity (Figure 2). A similar pattern was found in the study of Kumar [39]. When the cement paste fluidity was 160 mm, the WPC reached 10.8 mm/s. In the case of the cement paste fluidity increasing to 200 mm, the WPC decreased by 39.8% to 6.5 mm/s. In principle, the WPC of porous mortar depends on its total void ratio, which could be suggested by Figure 3b. The correlation coefficient of WPC and the total void ratio reached 0.96, suggesting that a very good linear correlation existed between them and the WPC of the porous mortar increased with the increase in total void ratio.

### 4.2. Effect of Cement Paste Fluidity and Paste-to-Aggregate Ratio on the Properties of Porous Mortar Using RSM

The ANOVA results of the mathematical models are presented in Table 5. This table indicates that the models proposed for the compressive strength and WPC of porous mortar as a response from the independent variables paste fluidity and paste-to-aggregate ratio were of sufficiently high R^2^ values, indicating a high degree of correlation between the experimental and the predicted responses. However, not all terms (separate effect of PF, P/A and interaction terms) contributed equally to the response. The relative contributions of each term could be derived from the F-value and the significance of the contribution was evidenced by the *p*-values, i.e., significant if *p* ≤ 0.05.

The F-values 1.87 and 13.10 were obtained for 28-day compressive strength and WPC, respectively, indicating that the models were significant. There is only a 0.01 percent chance that such a large F-value could occur because of noise. Moreover, the desired result had an insignificant lack-of-fit, as indicated by a *p*-value greater than 0.05. As presented in Table 5, all the *p*-values obtained by ANOVA indicated that the lack-of-fit was not significant compared to the pure. 

**Figure 4:**
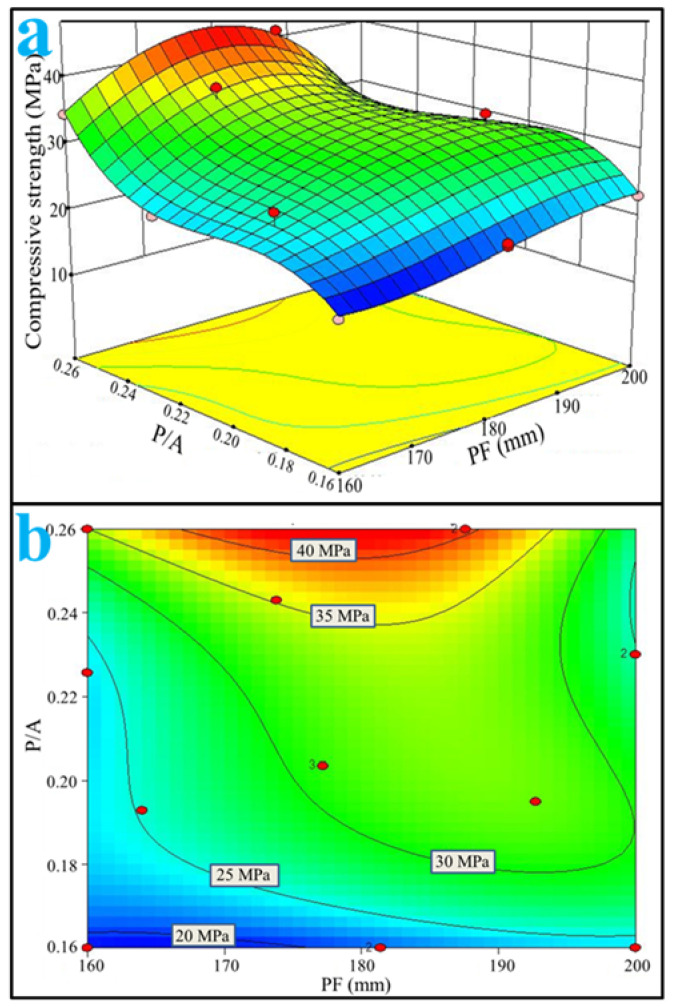
(**a**) Response surface and (**b**) contour line of 28-day compressive strength versus PF and P/A ratio.

The contour line of compressive strength versus cement paste fluidity and paste-to-aggregate ratio is presented in Figure 4b, which was determined by the response surface versus the same factors as described above (Figure 4a). From Figure 4b, at a given cement paste fluidity, the results indicated that the 28-day compressive strength significantly increased by increasing P/A from 0.16 to 0.26. The probable reason was that the aggregate coating thickness increased with the increase in paste-to-aggregate ratio. Moreover, the results in Figure 4 showed that porous mortar grades C20 (the 28-day compressive strength is not less than 20 MPa according to Chinese National Standards GB50107-2007, as follows), C25 and C30 could be designed by different paste-to-aggregate ratios while the range of cement paste fluidity was 160~200 mm. However, the mortar grade for C35 could only be designed by the paste-to-aggregate ratio of 0.24~0.26, and accordingly the range of cement paste fluidity was 160~194 mm. Similarly, mortar grade C40 could only be designed by the paste-to-aggregate ratio of 0.25~0.26 and the cement paste fluidity of 167~188 mm. The probable reason is that the high-grade porous mortar (i.e., C35 and C40) not only need an adequate aggregate coating thickness, but also an evenly distributed and uniform coating. 

**Figure 5:**
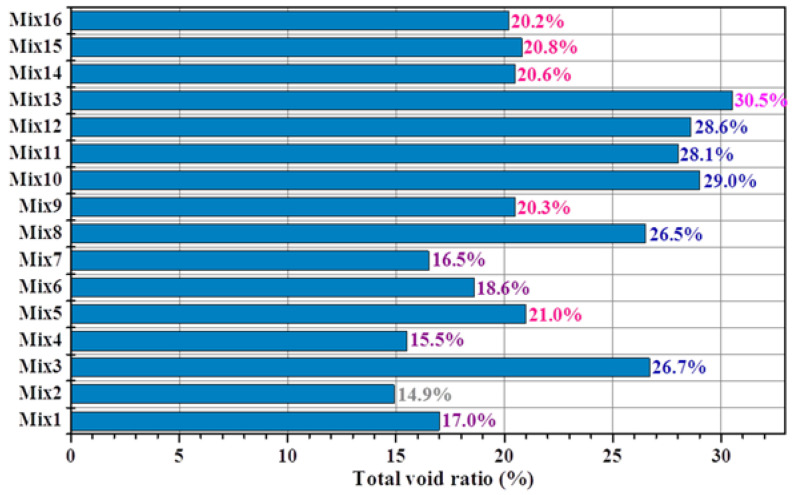
The total void ratios of porous mortar.

**Figure 6:**
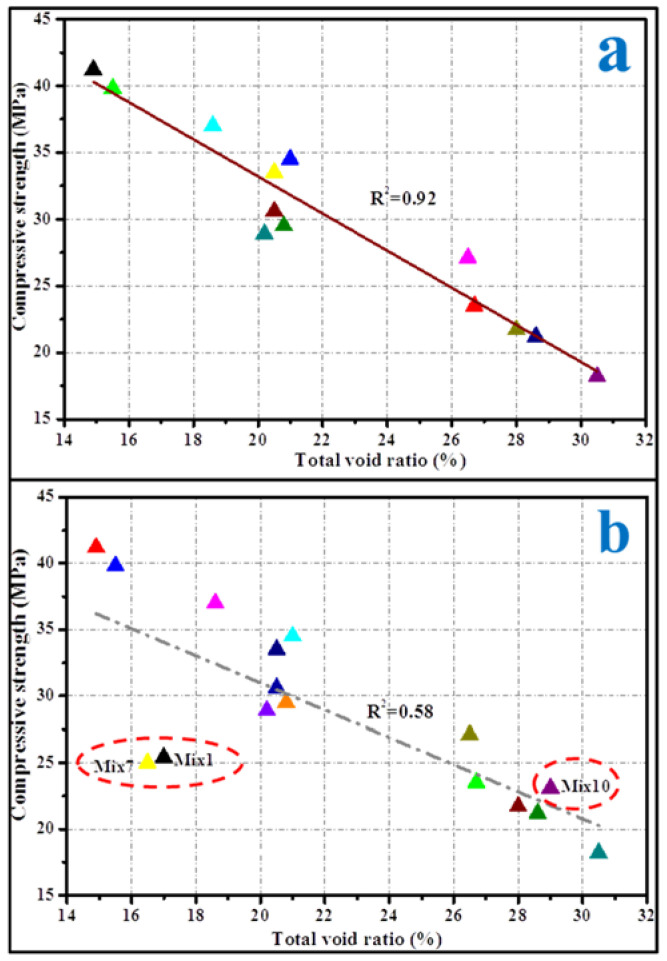
Correlation between total void ratio and 28-day compressive strength of porous mortar and each sample is represented by a colored triangle; the red circles represent groups of samples with significantly reduced correlations. (**a**) All designs in Table 4 except for Mix1, Mix7 and Mix10; (**b**) all designs in Table 4.

**Figure 7:**
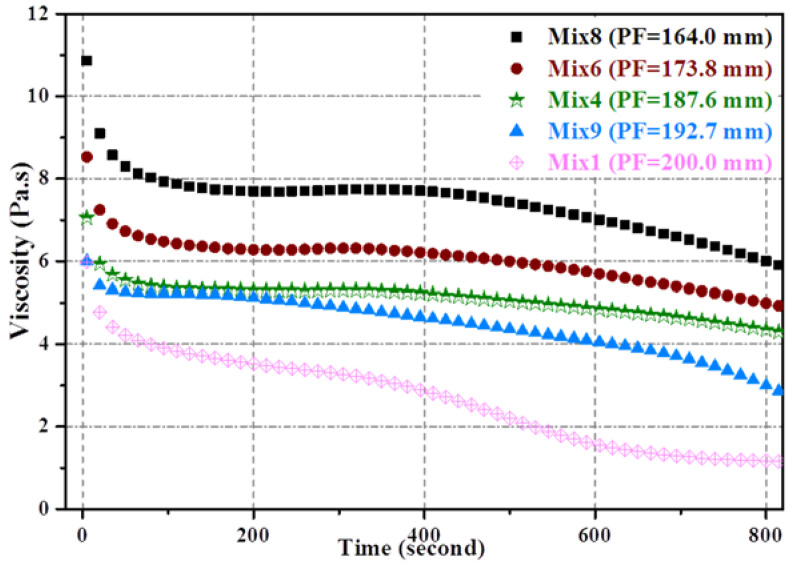
The viscosity of cement paste.

**Figure 8:**
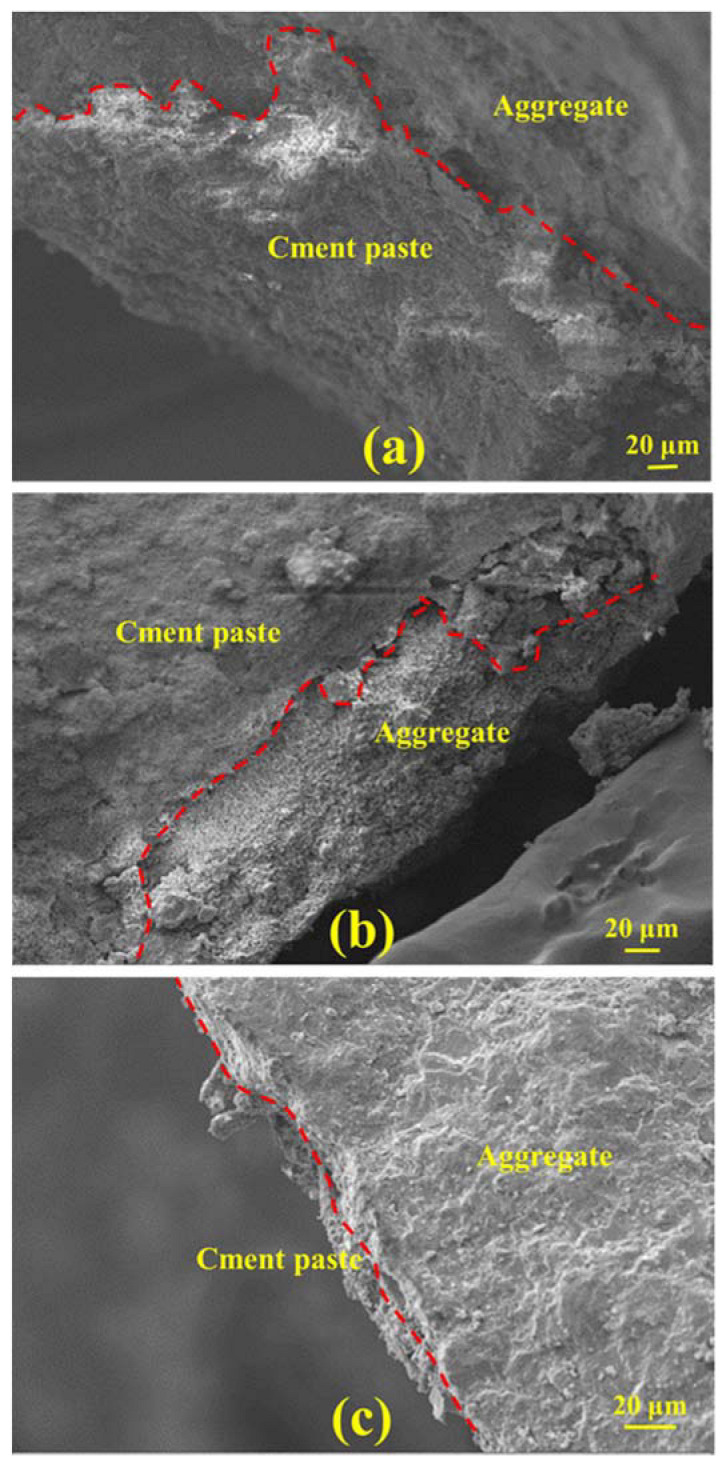
SEM images of the aggregate edge in porous mortar prepared with paste fluidities of (**a**) 160 mm, (**b**) 190 mm, and (**c**) 200 mm.

The total void ratios of the porous mortar (the mix designs are listed in Table 4) are shown in Figure 5, and the relationship between the total void ratio and 28-day compressive strength was established (Figure 6). When the PF was within 160–190 mm, there was a strong linear correlation between the total void ratio and the compressive strength of the porous mortar (R^2^ = 0.92) (Figure 6a). However, with the inclusion of mix designs with a PF of 200 mm (i.e., Mix1, Mix7 and Mix10), such correlation was significantly weakened (R^2^ = 0.58) (Figure 6b). It is likely that the viscosity of the cement paste decreased with the increase in cement paste fluidity (Figure 7); for instance, the beginning viscosity of Mix8 reached 10.8 Pa·s while that of Mix1 was only 4.7 Pa·s. Zhang, et al. [40] confirmed that the water film formed on the surface of the sand is one of the reasons for the change in its viscosity, and slurry with low fluidity has high viscosity. With such low viscosity, the cement paste was observed to have difficulty in adhering to the aggregate surface but accumulated at the bottom of the samples (Figure 8c). Figure 8 also presents SEM images of the aggregate edge in the porous mortar prepared at lower values of cement paste fluidity, as shown in Figure 8b. When the paste fluidity was too low, the coating layer on the aggregate surface was thick but non-uniform (Figure 8a). When the paste fluidity was increased to 190 mm, the paste was proved to be evenly covering the aggregate surface (Figure 8b). 

**Figure 9:**
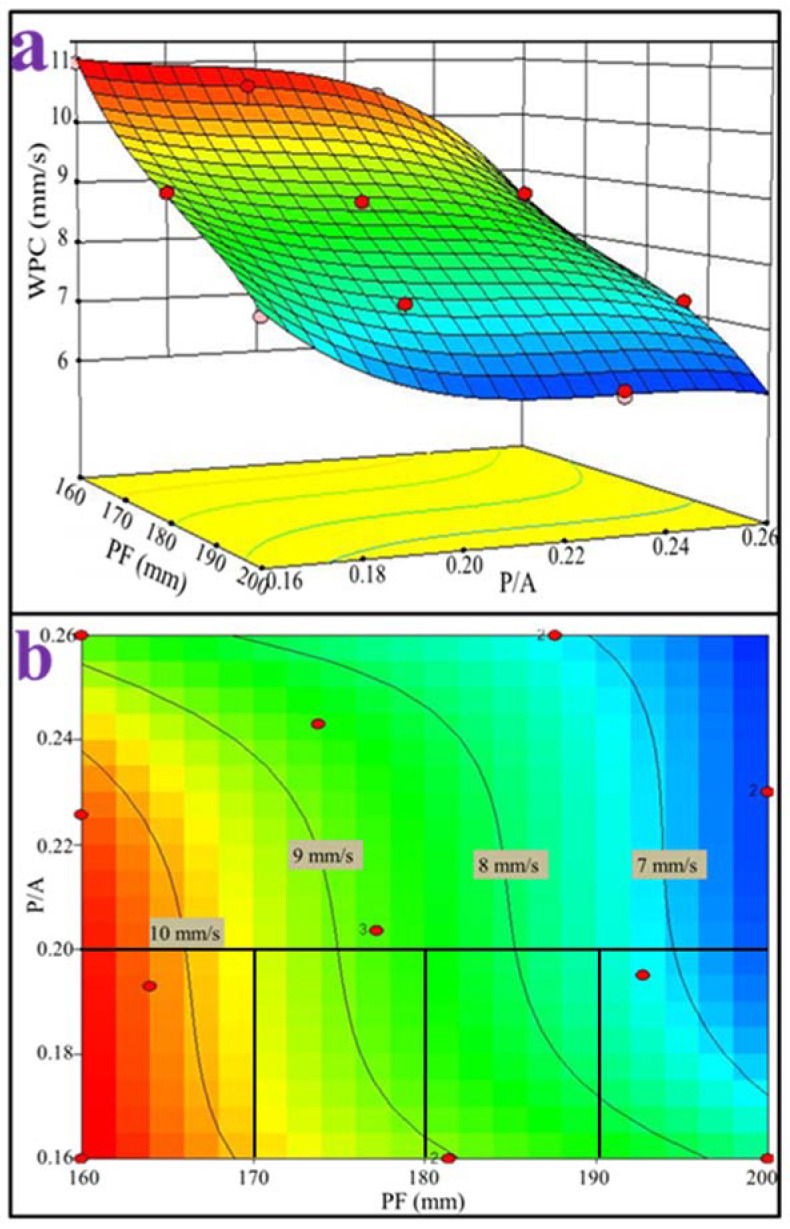
(**a**) Response surface and (**b**) contour line of WPC versus PF and P/A ratio. The red circles in the figure are experimental data points.

**Figure 10:**
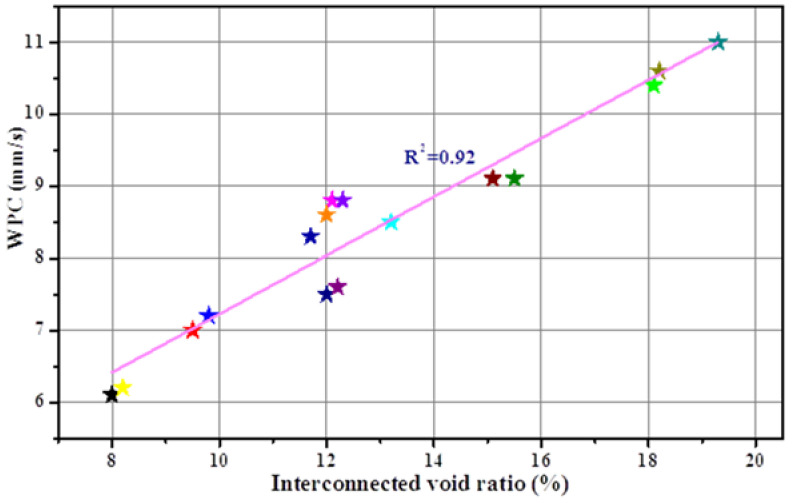
Relationship between interconnected void ratio and WPC of porous mortar and each sample is represented by a colored triangle.

**Figure 11:**
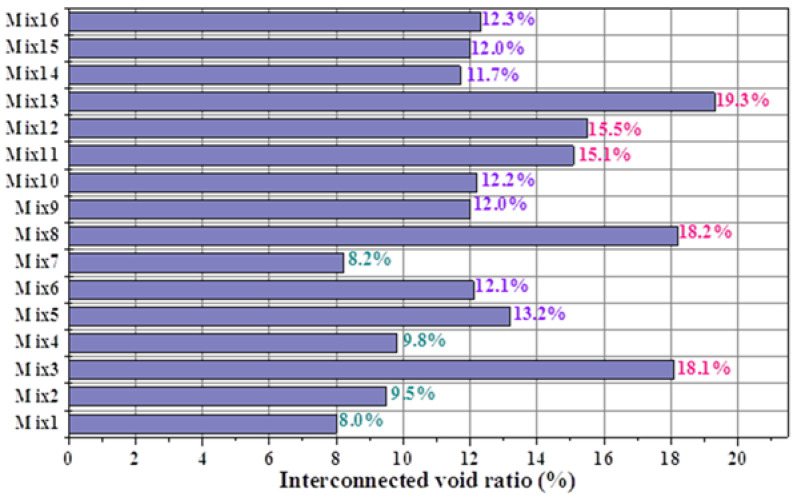
The interconnected void ratios of porous mortar.

**Figure 12:**
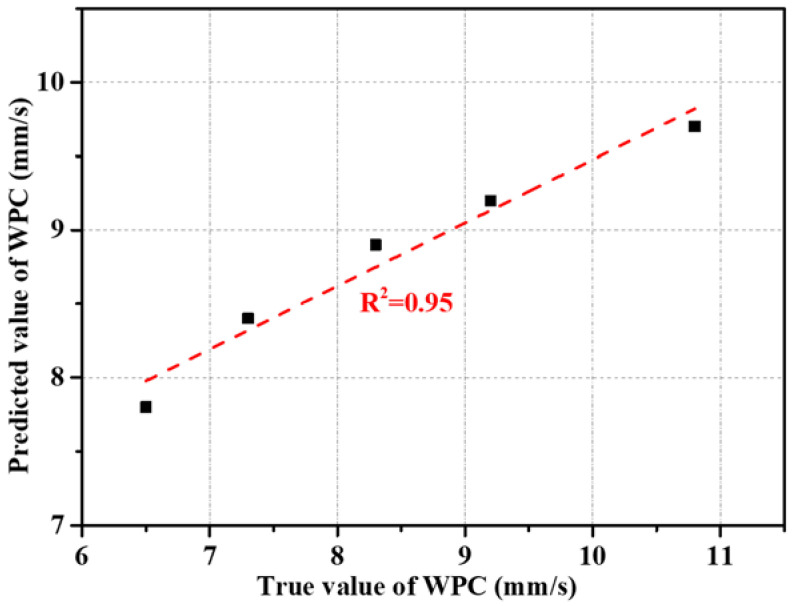
Correlation between measured WPC (MX1-5) and predicted WPC at PF of 160–200 mm and P/A of 0.20.

The contour line of the WPC versus cement paste fluidity and paste-to-aggregate ratio is shown in Figure 9b, and was determined by the response surface versus the same factors as illustrated above (Figure 9a). For a given paste-to-aggregate ratio, the WPC values decreased with increasing cement paste fluidity from 160 mm to 200 mm. This observation was not surprising given the highly correlated linear relationship between the interconnected void ratio and paste fluidity, as observed in Figure 1, and the linear correlation between the WPC and the interconnected void ratio, as shown in Figure 3b (PF as the single variable experimental results, correlation coefficient 0.96) as well as Figure 10 (PF and P/A double variables experimental results, correlation coefficient 0.92). The interconnected void ratio for the various designs in Table 4 is presented in Figure 11. At very low fluidity, the cement paste agglomerates easily during the mixing process due to its high viscosity, while at very high fluidity, the cement paste detaches easily from aggregates and accumulates at the bottom of the specimen. Therefore, the optimal cement paste fluidity and paste-to-aggregate ratio was vital for preparing a porous mortar with acceptable compressive strength and WPC. In order to balance the relationship between the two to the maximum, the use of the RSM method to establish a model for design optimization is widely accepted [41]. In order to verify the adequacy of the developed mathematical models, the results of the single factor experiment with P/A of 0.20 were plugged into the models. The predicted values of the WPC at PFs of 160, 170, 180, 190 and 200 mm and a P/A of 0.20 were calculated from the fitted model and are shown in Figure 12. In addition, the as-obtained WPC values were then compared with the measured data (Figure 12). The difference between the predicted value and the true value was less than 10%, and the points were well distributed along the 45-degree line, indicating the adequacy and reliability of the model and suggesting that the adequacy of the developed mathematical models was able to meet the requirements in this study.

Through RSM, one can easily estimate the optimal design mix using M-sand for the targeted property. For instance, a porous mortar of grades above C40 using M-sand could be designed at a P/A of 0.25–0.26 and a PF of 168–188 mm (Figure 4). It was also noted that the WPC of the porous mortar designed with a P/A of 0.25–0.26 and a PF of 168–188 mm could hardly reach more than 9 mm/s (Figure 9). Hence, the RSM, combining statistical and mathematical methods, proved to be a useful approach for multi-objective optimization in a porous mortar mix design with M-sand. 

### 4.3. Statistical Analysis of Responses

A statistical analysis was carried out to evaluate the experimental design in terms of the above-mentioned experiment results. A binary cubic model was obtained for prediction purposes. The veracity of the model as described above was evaluated based on the determination coefficients (i.e., R^2^) and standard deviation values (Table 5). The determination coefficients were measured as 0.9749 and 0.9840 for the 28-day compressive strength and WPC of the porous mortar material, respectively. There is a minimum determination coefficient of 0.80 for a good model fit, and a high value of R^2^ close to 1.00 demonstrates a desirable and reasonable agreement between the calculated and tested results. Furthermore, adequate precision was an additional index to evaluate the developed model. Adequate precision compares the range of the predicted values at the design points to the average prediction error. There is a minimum adequate precision of 4.00 for a good model fit. The adequate precision values of the models were 10.07 and 9.97 for 28-day compressive strength and WPC, respectively. The values of adequate precision are obviously greater than the minimum adequate precision and hence confirm that the model can be used to navigate the space defined by the central composite design.

## 5. Conclusions

The effect of cement paste fluidity (PF) on the properties of the porous mortar at a paste-to-aggregate ratio (P/A) of 0.20 was studied. The relationship between the responses (i.e., compressive strength and water permeability coefficient (WPC) of the porous mortar) and the two variables (PF and P/A) was established utilizing statistical analysis and the response surface method (RSM), and the accuracy of the model was analyzed. The main conclusions were drawn:

(1) At a fixed P/A of 0.20, with the increase in PF from 160 mm to 190 mm, the void ratio and WPC of the porous mortar decreased, while the compressive strength of the porous mortar increased. However, in the cases where the PF was more than 190 mm, the compressive strength dropped dramatically due to the detachment of the cement paste and its accumulation at the bottom of the porous mortar.
(2) The relationship models between the responses and the two variables were accurate, suggesting the RSM technique was a useful tool in optimizing the PF and P/A of porous mortar from manufactured sand (M-sand) with the desired properties as responses. The interaction of the two variables with the responses were visually observed by the 3D surface diagram, and the contour diagrams provided the intervals of the different responses.
(3) The WPC and mechanical strength are impacted by PF and P/A in a contradictive way, to some extent. The RSM could be utilized to set the goal parameters and design the experiment for porous mortar from manufactured sand based on the experimental outcome variables in order to reduce the design time, improve the performance of the existing process and product, improve reliability, and achieve product and process robustness.
(4) This paper made a systematic study of the effects of paste fluidity and paste-to-aggregate ratio on the properties of permeable mortar. In the future, the influence of the shape of M-sand on the mechanical properties, permeability, porosity and other basic physical properties, and the durability of permeable mortar will be investigated.

## Figures and Tables

**Table 1:** Chemical composition of OPC (wt.%).

Composition	CaO	SiO_2_	Al_2_O_3_	SO_3_	MgO	Fe_2_O_3_	K_2_O	TiO_2_	Los
OPC	60.19	20.57	5.58	3.8	2.59	2.59	1.3	0.3	1.7

**Table 2:** Physical properties of M-sand.

Properties	Gradation (mm)	Apparent Density (kg/m^3^)	Water Absorption (%)	Bulk Density (kg/m^3^)
Results	2.5–5	2425	2.49	1625

**Table 3:** Mix proportions of porous mortar.

Mixture No.	W/C ^a^	P/A ^b^	PF ^c^ (mm)
MX1	0.26	0.20	160
MX2			170
MX3			180
MX4			190
MX5			200

Note: ^a^: W/C—water-to-cement ratio; ^b^: P/A—paste/aggregate ratio; ^c^: PF—paste fluidity.

**Table 4:** Mixture design and experimental results.

Mixture No.	Mixture Design			Responses	
	W/C	PF (mm)	P/A	28-Day Compressive Strength (MPa)	WPC (mm/s)
Mix1	0.26	200.0	0.23	25.4	6.1
Mix2		187.6	0.26	41.2	7
Mix3		160.0	0.23	23.5	10.4
Mix4		187.6	0.26	39.8	7.2
Mix5		160.0	0.26	34.5	8.5
Mix6		173.8	0.24	37.0	8.8
Mix7		200.0	0.23	25.0	6.2
Mix8		164.0	0.19	27.1	10.6
Mix9		192.7	0.20	33.5	7.5
Mix10		200.0	0.16	23.1	7.6
Mix11		181.4	0.16	21.7	9.1
Mix12		181.4	0.16	21.2	9.1
Mix13		160.0	0.16	18.2	11
Mix14		177.2	0.20	30.6	8.3
Mix15		177.2	0.20	29.5	8.6
Mix16		177.2	0.20	28.9	8.8

**Table 5:** ANOVA results for fitted numerical models.

	28-Day Compressive Strength				WPC			
Source of Data	Sum of Squares	df	F-Value	*p*-Value > F-Value	Sum of Squares	df	F-Value	*p*-Value > F-Value
Model	692.50	9	25.94	0.0004	31.42	9	41.06	0.0001
A-PF	5.55	1	1.87	0.2202	1.11	1	13.10	0.0111
B-P/A ratio	2.55	1	0.86	0.3899	0.014	1	0.17	0.6943
AB	26.67	1	8.99	0.0240	0.11	1	1.33	0.2919
A^2^	78.89	1	26.60	0.0021	3.588 × 10^−4^	1	0.00422	0.9503
B^2^	0.025	1	0.00833	0.9302	0.037	1	0.43	0.5349
A^2^ B	44.85	1	15.12	0.0081	0.056	1	0.66	0.4485
AB^2^	8.86	1	2.99	0.1346	0.56	1	6.62	0.0422
A^3^	0.96	1	0.32	0.5901	0.042	1	0.49	0.5082
B^3^	25.51	1	8.60	0.0262	0.13	1	1.47	0.2704
Residual	17.80	6			0.51	6		
Lack of fit	15.13	1	28.31	0.8531	0.36	1	11.82	0.1185
Pure error	2.67	5			0.15	5		
R^2^	0.9749				0.9840			
Adj. R^2^	0.9374				0.9601			
AP	10.07				9.97			

## Data Availability

Data available on request from the authors.
